# Knowledge, Attitude and Practice of Mothers in the Management of Diarrhoea among Under-five Children in a Rural area of India

**DOI:** 10.4314/ejhs.v34i4.8

**Published:** 2024-07

**Authors:** Kayala Venkata Jagadeesh, Ashwini Narasannavar, Mahantesh Kamble, S Prakasha, Bandaru Yeswanth Raja

**Affiliations:** 1 Department of Public Health, J N Medical College, KLE Academy of Higher Education and Research, Belagavi, Karnataka, India; 2 Department of Pharmacy Practice, KLE College of Pharmacy, *KLE Academy of Higher Education and Research*, Belagavi, Karnataka, India

**Keywords:** Diarrhoea, Mothers, under-five children, IDCF, knowledge, attitude, practice

## Abstract

**Background:**

Diarrhoea is defined as the passage of loose, liquid or watery stools more than three times a day. Though simple and effective treatment measures are available which can markedly reduce diarrhea associated morbidity and mortality, yet in developing countries due to poor diarrhea and Oral rehydration therapy (ORT) related knowledge, diarrhoea still continues to be a major public health problem. The study aimed to estimate the knowledge, attitude and practice of mothers in the management of diarrhoea among under-five children in a rural area of India.

**Methods:**

A Cross-sectional study was conducted among mothers having children below five years of age in rural area of India. Proportionate sampling technique was used to recruit mothers. A pretested, pre designed structured questionnaire was used to obtained the data.

**Results:**

A total of 382 mothers were participated in the study. The mean age of study participants was 25.72 ± 3.98 years. Overall knowledge score showed that (74.6%) three-fourth of the study participants had average knowledge regarding diarrhoeal management. Majority of the mothers (62.6%) showed positive attitude in diarrhoea management The practice scores showed that nearly half of the study participants (50.3%) had good practice regarding diarrhoea management.

**Conclusion:**

The knowledge of mothers regarding management of diarrhoea among under-five children was average and most mothers had positive attitude. However, only half of the mothers had good practice.

## Introduction

Diarrhoea is defined as the passage of loose, liquid or watery stools more than three times a day (1, 2). It is treated as a serious health problem worldwide. It is perhaps one of the most important causes of sickness and death among infants and children in developing countries. Though simple and effective treatment measures are available which can markedly reduce diarrhea-associated morbidity and mortality, due to poor diarrheal and Oral rehydration therapy (ORT) related knowledge in developing countries, diarrhoea still continues to be a major public health problem (3,4).

Worldwide, diarrhoea claims the life of 2 million children each year (5). It is the second most common cause of mortality and morbidity among children, following acute respiratory infections (6,7,8) Particularly, the burden of diarrhoeal morbidity prevails more in developing countries like India, accounting for three episodes per year in children below three years of age (5,6). According to WHO, diarrhoea is the third most common cause of death in under-five children in India (9, 10). Around 1.2 lakhs of children die due to diarrhoea annually in the country and the worst affected are children from poor socioeconomic status (11). Similarly, one out of five children worldwide who die of diarrhoea is from India (12).

In 1981, India launched the Diarrhoeal Disease Control Programme to overcome the high disease burden, wherein the focus shifted to strengthening the case management of diarrhoea in children under five years of age (13). Later, it was integrated with child survival and safe motherhood (CSSM). Diarrhoeal mortality has been reduced in under-five children for the past two decades due to case management through oral rehydration therapy, as per standard guidelines recommended by WHO (2,14).

Childhood diarrhoea continues to be a major killer disease among under-five children in many states, contributing to 10% of under-five deaths in the country. One of the goals of the Millennium development goals and the National Health Mission (NHM) is to increase efforts to reduce childhood mortality. In 2014, India introduced Intensified Diarrhoea Control Fortnight (IDCF) program intending to attain zero deaths due to childhood diarrhoea. It has a set of activities: intensification of advocacy & awareness generation activities for diarrhoea management, strengthening service provision for diarrhoea case management, the establishment of ORS-Zinc corners, prepositioning of ORS by ASHA to households with under-five children and awareness generation activities for sanitation & hygiene (6, 11). ORS is the non-propriety name for balanced glucose and electrolyte mixture, recommended and approved by UNICEF and WHO to prevent dehydration globally (7). It was estimated that 60-70% of deaths related to diarrhoea are caused by dehydration. Thereafter, ORT has revolutionized the concept of diarrhoea management (15). ORT has prevented more than 50 million child death due to diarrhoea over the past 25 years (7). Thus, the focus of IDCF is on the delivery of simple proven interventions that have a large impact on the control of childhood diarrhoeal morbidity and mortality (11).

Diarrhoeal episodes are mostly self-limiting, however, deaths are due to severe dehydration (16, 17). Management of diarrhoea can be done at both primary and secondary prevention levels. Primary prevention includes immunization, improvement of sanitation, exclusive breastfeeding and water quality. Secondary prevention includes early recognition of dehydration due to diarrhoea and prompt oral rehydration therapy through ORS or homemade fluids, increased breastfeeding, Zinc therapy and use of appropriate antibiotics for severe diarrhoeal cases (18).

The new recommendation of WHO includes two recent advances- demonstration of the increased efficacy of a new ORS formulation, containing lower glucose and salt levels, and zinc supplementation with ORS in the management of diarrhoeal disease. The new ORS formulation is packaged in a sachet to be dissolved in one litre of clean water. Zinc is available as 10 mg or 20 mg rapidly disintegrating tablets which can be administered using a teaspoon of clean water or expressed breast milk to children of age 1-59 months. It reduces diarrhoeal episodes and severity. Usually, Zinc is recommended for 10-14 days which lower diarrhoea incidence in the following 2 to 3 months (19). Lack of awareness about ORS can lead to improper utilization of health services available in the community.

Childhood diarrhoea management can be improved significantly through health education for the mother (2 Mothers can reduce morbidity and mortality of under-five children by adopting healthy practices. As mothers are considered, primary health care providers, their knowledge regarding diarrhoea causes, signs and symptoms, prevention and control is very essential to achieve zero mortality due to diarrhoea among under-five children (14, 15). However, many studies revealed that caregivers were providing high or low osmolar ORT mixture to their children because of a lack of knowledge on how to prepare ORS correctly (20). Thus, this study was planned to assess the knowledge, attitude and practice of mothers in the management of diarrhoea among under-five children after Implementation of IDCF Program in a rural area of India.

## Materials and Methods

A Cross-sectional study was conducted for one year in a rural area of the Belagavi District.

**Study settings**: Study area is located in Belagavi district having one PHC covering about 70,000 population. 9 subcenters are working under it which in turn, constitute 74 Anganwadi centres covering all the population.

**Study population**: Mothers having under-five children in the study area were included. Those mothers who were reluctant to give consent and residing in the study area for less than 1 year were excluded from the study because the IDCF program has been implemented on yearly basis for a fortnight and also to avoid recall bias. For the preparation of the population frame, initially, at the PHC level, we had collected the list of Anganwadi centres present under each sub-centre. Later, the list of mothers was obtained from each Anganwadi centre.

**Sample size**: The sample size was calculated by using the formula Z^2^pq/d^2^ at 95% C.I with Z value 1.96. It was estimated to 382 study participants.

**Sampling technique**: The current study used the Proportionate sampling technique to avoid selection bias in including the study population. So that the study population would be able to represent all the mothers under the PHC. Participants were selected based on the number of mothers covered by the respective Anganwadi centre. Thus, study participants were selected proportional to mothers covered under the Anganwadi centre.

At each Anganwadi centre, systematic random sampling was done to recruit mothers for our study. For this, initially, we calculated the random interval/every nth sample to be selected from the list of mothers collected at the Anganwadi centre. We took the help of local ASHAs to find out the household of respective mothers in the population frame.

**Data collection tools and procedure**: A pretested, pre-designed structured questionnaire was used to obtain the data. Questionnaire was prepared based on the IDCF guidelines (11) especially questions related to knowledge and practice. For designing attitude questionnaire, the study used published literature from western Ethiopia (5) and IDFC guideline as well. A pilot study was conducted on 10% of the sample size to test the reliability of the questionnaire. Data collectors were trained for the above questionnaire and allowed to collect the data under the supervision of the principal investigator. Data collectors were selected in such a way that they are good at Kannada, Marathi and Hindi. Being a border district of Karnataka to Maharashtra, the study population was multilingual, knowing either Kannada or Marathi or Hindi.

The process for data collection involved a systematic approach where data collectors primarily contacted local Anganwadi workers on every day of data collection in respective sub-centres of the study area. They played a crucial role in identifying the selected mothers in accordance with the sampling technique employed for the study. This collaboration ensured that the data collection targeted the appropriate individuals, aligning with the study's methodological requirements. Once the mothers were identified in the population frame, a house-to-house visit had been made and mothers were interviewed using a pretested, predesigned questionnaire. Parts of the questionnaire included socio-demographic profile, knowledge, attitude and practice questions. Modified BG Prasad socio-economic status scale (2017) has been used to categorize study participants into different social classes. Five Likert scale was used for the attitude questionnaire. Utmost care has been taken to maintain privacy and confidentiality.

**Outcome variables**: Ascertainment of overall knowledge, attitude and practice of study participants regarding the management of diarrhoea among under-five children was performed. According to the score obtained, knowledge groups were categorized into good, average and poor knowledge. Attitude groups were classified into positive attitude and negative attitude. Practice groups include good practice and bad practice.

**Statistical analysis**: Data was entered in excel and analyzed using SPSS version 20.0. Results were expressed as frequency and percentages. Descriptive statistics i.e., Mean and standard deviation were used to describe the mean age and average number of children of the study population. Scoring was done by giving 1 for the correct and 0 for the wrong answer. Overall scoring was calculated for knowledge, attitude and practice. Multiple response questions were excluded from scoring. Chi-square and ANOVA were used to estimate the association between socio-demographic characteristics and outcome variables and within outcome variables.

**Ethical issues**: Ethical clearance was obtained from JNMC Institutional Ethics Committee. The written informed consent form was translated into local languages including Kannada, Marathi and Hindi. Prior to data collection, study details were explained to each participant and were told that they had the right to discontinue from the study at any point of time, if they were not willing to respond to questions. Inform consent was obtained; a copy of the informed consent document was given to each study subject.

## Results

**Socio-demographic characteristics**: Socio-demographic characteristics of study participants are presented in Table 1. Out of 382 mothers participated in the study, majority 313 (81.9%) were in the age group of 21-30 years, and the mean age of study participants was 25.72 years with standard deviation (SD) of 3.98 years. Concerning number of children, 175 (45.8%) mothers reported that they have two children, and the mean of number of children for participants were 1.82 with SD of 0.77. Regarding religion of study participants, majority 328 (85.9%) were Hindus.

**Table 1:** Socio-demographic characteristics of the study participants

Variable	Study participants (n=382) N(%)
**Age group (in years)**	
≤ 20	40(10.5)
21-30	313(81.9)
31-40	27(7.1)
≥ 41	2(0.5)
**Number of children**	
One	144(37.7)
Two	175(45.8)
≥ Three	63(16.5)
**Religion**	
Hindu	328(85.9)
Muslim	53(13.9)
Others	1(0.3)
**Social class**	
Upper class	10(2.6)
Upper middle class	46(12.1)
Middle class	75(19.6)
Lower middle class	83(21.7)
Lower class	168(44.0)
**Level of education**	
Primary	72(18.8)
Secondary	190(49.7)
PUC	74(19.4)
Graduate and above	24(6.3)
Illiterate	22(5.8)
**Source of water**	
Bore well and Dug well	85(22.3)
Tap water	288(75.4)
Tube well	9(2.4)

**Knowledge of study participants regarding management of diarrhoea among under-five children**: Table 2 represents the knowledge of mothers regarding diarrhoea management. 360(94.2%) had given a correct definition for diarrhoea. More than half 206 (53.9%) of the mothers had knowledge regarding signs and symptoms of diarrhoea. Regarding ORS, 311 (81.4%) of the study participants had knowledge. Concerning ORS preparation, 257(67.3%) of the study participants had knowledge. According to this study, only 7(1.8%) of study participants had knowledge regarding importance of zinc supplementation during diarrhoea. We also investigated about what should be done if child is suffering from diarrhoea, 95.5% reported that they should consult physician, 3(0.8%) of them stated that they stay in home, 14(3.7%) of the study participants stated that administer self-medication (Not presented in Table 2). Overall knowledge score showed that (74.6%) three-fourth of the study participants had average knowledge regarding diarrhoeal management (Graph1).

**Table 2:** Knowledge regarding management of diarrhoea as stated by study respondents

Variables	Yes (%)	No (%)
Correct definition given	360 (94.2)	22 (5.8)
Correct Signs and Symptoms of diarrhoea reported.	206 (53.9)	176 (46.1)
Know about ORS	311 (81.4)	71 (18.6)
Know the preparation of ORS	257 (67.3)	125 (32.7)
Know about Zinc Importance during Diarrhoea	7 (1.8)	375 (98.2)
Know about government is supplying Free ORS & Zinc through ASHA	7 (1.8)	375 (98.2)

The majority 328 (85.9%) of the study participants had no knowledge regarding danger signs of diarrhoea, whereas few identified as Lethargy (8.9%), Thirsty and Dry mouth (8.15%), followed by severe dehydration (1.3%), not able to drink (1.0%), sunken eyes (0.5%), unconscious (0.5%) and Loss of Skin Stretchiness (0.3%). Table 3 shows the knowledge of study participants regarding amount of water required for ORS preparation. Among 257 participants who were aware of ORS preparation, 76(29.6%) had knowledge regarding correct amount of water required for ORS preparation.

**Table 3:** Knowledge regarding amount of water required for ORS preparation

Response to water required	Frequency (%)
Correct	76(19.9)
Wrong	158(41.4)
Don't Know	23(6.0)
Don't know ORS preparation	125(32.7)
Total	382(100.0)

**Fig 1:**
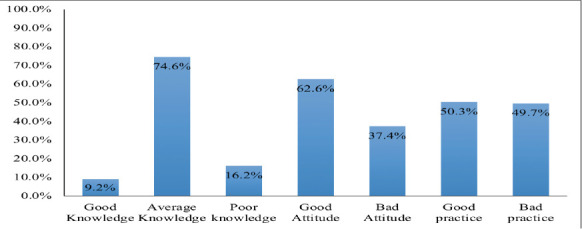
Overall Knowledge, Attitude and Practice of mothers in the management of Diarrhoea among under-five children

**Attitude of mothers towards management of diarrhoea among under-five children**: Table 4 represents the attitude of mothers towards diarrhoeal management in under-five children. Overall attitude score revealed that majority (62.6%) of the study participants had positive attitude towards diarrhoeal management (figure 1).

**Table 4:** Attitude of study participants towards management of diarrhoea among under-five children

Variables	Strongly Agree (%)	Agree (%)	Neutral (%)	Disagree (%)	Strongly Disagree (%)
Diarrhoea attacks mostly in bottlefed children	74(19.4)	100(26.2)	38(9.9)	167(43.7)	3(0.8)
Diarrhoea is a disease of the poor	17(4.4)	66(17.3)	67(17.5)	218(57.1)	14(13.7)
Diarrhoea is a killer disease	119(31.2)	120(31.4)	25(6.5)	109(28.5)	9(2.4)
Diarrhoea is a problem in the community	125(32.7)	203(53.2)	8(2.1)	44(11.5)	2(0.5)
Teething in children causes diarrhoea	305(79.8)	65(17.0)	6(1.7)	6(1.7)	0(0)
Diarrhoea is a curable disease	207(54.2)	167(43.7)	5(1.3)	3(0.8)	0(0)
Child's/infant's faeces is hazardous to health	46(12.0)	110(28.8)	93(24.4)	130(34.0)	3(0.8)
Liquid food aggravates diarrhoea	109(28.5)	58(15.2)	54(14.2)	154(40.3)	7(1.8)
Continuation of breastfeeding is important during diarrhoea	189(49.5)	145(37.9)	0(0)	43(11.3)	5(1.3)
Oral rehydration salts solution cures diarrhoea	90(23.6)	173(45.3)	57(14.9)	57(14.9)	5(1.3)

**Diarrhoea management Practice of mothers among under-five children**: Table 5 shows that practice of study participants regarding diarrhoeal disease management among under-five children. First three questions of practice were not applicable to participants who responded No for ORS preparation under knowledge questionnaire. A total of 166 (43.5%) study participants had never used ORS for diarrhoea management to their children. Overall practice scores showed that nearly half of the study participants (50.3%) had good practice regarding diarrhoea management (Graph 1).

**Table 5:** Practice of study participants regarding management of Diarrhoea among under-five children

Variables	Response category	Frequency (%)
Correct technique of ORS preparation^a^	Yes	72 (18.8)
	No	144 (37.7)
Hand washing before ORS Preparation^a^	Yes	202 (52.9)
	No	14 (3.6)
Fluid for ORS^a^	Boiled and Cooled water	188 (49.2)
	Normal Water	28 (7.3)
Washing hands before Handling food	Yes	369 (96.6)
	No	13 (3.4)
Hand wash using	Dettol	99 (25.9)
	Soap	225 (58.9)
	Normal Water	58 (15.2)
Zinc along with ORS	Yes	06 (1.6)
	No	376 (98.4)
Breastfeeding during diarrhoea	Yes	339 (88.7)
	No	43 (11.3)
Bottle feeding	Yes	122 (31.9)
	No	260 (68.1)

a Represents mother's responses who knew ORS preparation

After estimating overall knowledge, attitude and practice, the study participants were categorized into three knowledge groups (Good, Average and Poor), two attitude groups (Positive and Negative) and two practice groups (Good and Bad). As far as overall scores are concerned, there is a significant difference in attitude and practice of mothers regarding management of diarrhoea among under-five children in three different knowledge groups (F= 11.639; p=0.000). Atleast women with average knowledge showed better practice and positive attitude in diarrhoea management. It is also found that there is a significant difference in the practice of mothers regarding diarrhoeal management among under-five children between two attitude groups (positive and negative attitude) (p=0.000) showing good practice among positive attitude group.

*Association between socio demographic characteristics of mothers and their Knowledge, attitude and practice:*

There is an association between socio-demographic characteristics between knowledge, attitude and practice of mothers in the management of Diarrhoea. It was found that there was a significant association between knowledge of mothers and number of children (F=3.396, p=0.035). There was a significant difference in the attitude of mothers among socio-economic groups regarding diarrhoea management (*χ*^2^ = 10.380, p=0.034). The practice of mothers was also found associated with their education (*χ*^2^ = 10.761, p=0.029) and number of children (*χ*^2^ = 8.147, p=0.017).

## Discussion

### Socio-demographic details

In this study, majority of mothers were in age group between 21-30 years. Similar study conducted in Kalaburgi, Karnataka revealed that majority of the participants were between age group of 21-25 years (6). In this study, 85.9% of participants were Hindus similarly, another study conducted in Kancheepuram, Tamilnadu reported majority of the study population as Hindus (48%) followed by Christians (42%) and Muslims (10%) (8). In the current study, 44.0% belonged to lower class, followed by Lower middle class (21.7%), Middle class (19.6%), upper middle class (12.1%) and upper class (2.6%). Whereas in a study conducted among mothers in Tamil Nadu reported that 32% of study participants were in middle class followed by other income classes (8). In our study, nearly half of the study participants had education up to secondary level, and only 5.8% were illiterates. In contrary, another study conducted in Karnataka reported that majority (37.25%) of the mothers were illiterates followed by secondary education level (20.09%) (6).

### Knowledge regarding management of diarrhea

In this study, 94.2% participants were aware of Diarrhoea definition, which is in contrary with the results of another study conducted in Bangalore, reported that only 46% of mothers had baseline knowledge regarding diarrhoea definition (4). In the present study, more than half (53.9%) of the mothers had knowledge regarding signs and symptoms of diarrhoea. Our findings are similar to study conducted in Nepal among mothers inquiring about signs and symptoms of diarrhoea as loose motions and pain abdomen (21).

In this study, 85.9% of the study participants had no knowledge regarding danger signs of diarrhoea. A study in Western Ethiopia reported that only 9% were not aware of dangers signs of diarrhoea which was contradicting the results of our study (5). One more study conducted in Rajasthan reported that 8.5% of mothers were aware of at least one danger sign (18). In this study, 81.4% of the mothers had knowledge regarding ORS and 67.3% had knowledge regarding correct technique of ORS preparation. Similar results were observed in study conducted in Karnataka where 86.27% of mothers were aware of ORS sachets and 58.52% were aware of correct preparation (6).

Regarding free ORS & Zinc supply through ASHA workers by government, only 1.8% study participants knew about it. It is one of the strengths of current study that no literature has found discussed about the knowledge of mother regarding free ORS & Zinc supply by government through ASHA workers. In the present study, among 257 participants who were answered that they were aware of ORS preparation, only 29.6% had knowledge regarding correct amount of water required for ORS preparation. Another study conducted in Maharashtra reported similar findings that 37.8% mothers aware about correct amount of water required for mixing ORS (3). These findings were comparatively less than reported by other two studies carried out in Lahore (22,23). That infers that there is a need of hour to educate mothers in the study area about ORS preparation.

In this study, only 1.8% had knowledge regarding importance of zinc supplementation during diarrhoea. Another study conducted in India reported that no study participant were aware of zinc (13). It reflects that there is a huge lacunae found in WHO recommendation of Zinc supplementation for management of diarrhea (19).

### Attitude of mothers towards management of diarrhea

One of the strengths of this study were that it was that only one study which has reported attitude of mothers using a 5-Likert scale and also estimated overall attitude. Regarding attitude towards management of diarrhoea, 43.7% of mothers disagreed that diarrhoea attacks mostly bottlefed children. Similar findings reported in a study at Western Ethiopia that 54.7% disagreed in response to statement diarrhoea attacks mostly bottlefed children (5). In the present study, 31.2% mothers strongly agreed that diarrhoea is a killer disease. In contrast, 82.3% of study participants in Western Ethiopia agreed that it is a killer disease (5).

In this study, 79.8% mothers strongly agreed that teething cause diarrhoea. Similar results were reported by a study conducted on mothers in Western Ethiopia as 70.7% agreed that teething causes diarrhea (5). In this study, 49.5% of the study participants strongly agreed and 37.9% agreed that mothers should continue breastfeeding during diarrhoeal episode. Another study conducted among mothers in Tamil Nadu reported higher prevalence of attitude about 98% towards breastfeeding (8). Results of current study about a statement that ORS cures diarrhea (23.6% strongly agreed and 45.3% agreed) is comparatively less than study conducted in Tamil Nadu however, apparently closer to findings of a study in Western Ethiopia (5).

Our study findings showed that overall attitude score of study participants showed positive attitude towards management of diarrhea. Another study conducted among mothers of under-five children in India also reported that they had shown a positive attitude towards the use of oral rehydration solution in the management of diarrhea (8).

### Practice of mothers in the management of Diarrhoea

With regards to practice, current study had considered correct practice of ORS preparation only if participants had used boiled and cold water and done hand wash before ORS preparation, as it was recommended in IDCF program and it was found that only few mothers followed correct technique (11). In contrary, a study conducted in Lahore reported that 76% of mothers had prepared ORS correctly (22). Similarly another study conducted in India reported that 90% mothers were using boiled water for mixing ORS and 90% of mothers had washed their hands before preparation (8).

One of the unique characteristics of this study are that we collected data on zinc administration along with ORS, majority of the mothers (98.4%) did not use zinc for diarrhoea management showing a gap in the recommendations of WHO and IDCF program (11,19). No past study found reported practice of Zinc administration with ORS during diarrhoea. Regarding breastfeeding children during diarrhoeal episode, our study findings (88.7%) were apparently more than reported by a study conducted in Bankura where 79.6% of mothers were continuing breastfeeding to their children during diarrhoea attack (16). This was showing a good practice concerning breastfeeding (considering during diarrhoeal attack) in the study area. Practice of breastfeeding was little lesser than findings reported by a study conducted in Tamil Nadu where 94% of mothers had continued breastfeeding even child was on ORS (8). Our study findings include practice of washing hands before handling food which is an important factor to prevent diarrhoea among children. We also probed mothers to answer what do they use for hand wash? Majority were using soap followed by Dettol and normal water.

### Association between socio-demographic characteristics and overall knowledge, attitude and practice of mothers

This study affirmed an association between socio-demographic profile and knowledge, attitude and practice of mothers in the management of Diarrhoea. Education level of mothers was significantly associated with their practice regarding management of diarrhoea. There was a significant difference in the practice levels of mothers among different knowledge groups. As majority of mothers showed average knowledge, it is important to increase the knowledge of mothers on management of diarrhoea. Knowledge had impact on attitude of mothers which in turn affects their practice. It was also found that there was a strong association between knowledge on Zinc importance and practice of using zinc along with ORS during diarrhoeal episode. Through this study, we append association between knowledge, attitude and practice of mothers in the management of Diarrhoea among under-five children to the existing literature.

In conclusion, over all knowledge of mothers regarding management of diarrhoea among under-five children was found to be average. Particularly, knowledge regarding zinc importance during diarrhoea was negligible, even though WHO and IDCF program recommended Zinc therapy in the management of diarrhoea. Over all, we found a positive attitude of mothers towards management of diarrhoea among under-five children. However, only half of the mothers had good practice. Unfortunately, most of the participants had never used ORS and negligible number of mothers had administered zinc along with ORS.

It is recommended to evaluate Intensified Diarrhoea Control Fortnight (IDCF) program across the country. However, Mothers have positive attitude towards management of diarrhea, there is a gap in the practice towards management which is needed to be identified and addressed. It would also be better to conduct repeated IEC activities to improve the knowledge of mothers about diarrhoea management in under-five children using ORS and Zinc. Home-made preparation of ORS shall be taught to mothers by Medical officers, ANMs, and ASHA.

This study is confined to only one PHC in Karnataka due to limited time and resource available, so, results can be generalized to population under same PHC only. Practice of anti diarrhoeal mediation was not considered in the current study. Amount and frequency of ORS should be administered for children during diarrhoeal episode was not captured.
